# Cross-sectional and longitudinal associations between anxiety and acoustic-prosodic markers in adolescents

**DOI:** 10.1017/S0033291725102274

**Published:** 2025-10-28

**Authors:** Silvia Ciampelli, Janna de Boer, Sanne Koops, Alban Voppel, Hugo Corona Hernandez, Albertine Oldehinkel, Iris Sommer

**Affiliations:** 1 University of Groningen: Rijksuniversiteit Groningen, the Netherlands; 2 Universite McGill: McGill University, Canada

**Keywords:** adolescents, anxiety disorders classification, machine learning, predictive modelling, speech markers

## Abstract

**Background:**

Adolescence marks a critical period for the onset of anxiety disorders, yet they frequently remain undiagnosed due to barriers such as reluctance to self-disclose symptoms. Objective screening methods that bypass self-report may improve early detection. Speech-derived acoustic markers have emerged as a promising avenue for identifying anxiety disorders. This study investigates associations between acoustic properties of speech, anxiety severity, and anxiety diagnoses in adolescents, evaluated cross-sectionally and longitudinally.

**Methods:**

Speech samples from 581 adolescents were collected during the Trier Social Stress Test. Acoustic features were extracted using OpenSMILE and analyzed for cross-sectional associations with anxiety severity (Spearman’s correlations) and longitudinal predictions of future anxiety (linear regressions). Random forest (RF) classifiers with 10-fold cross-validation were used to classify anxious and healthy individuals using acoustic features. Analyses were stratified by sex.

**Results:**

RFs achieved the highest performance for the longitudinal classification of social anxiety disorder (SAD), with an AUC-ROC of 85% (males) and 74% (females). Adding acoustic features to baseline measures increased the variance explained in anxiety by 5.4% (males) and 10.9% (females). In males, higher anxiety was cross-sectionally correlated with reduced pitch slope, narrower pitch range, lower F1 frequency, and greater MFCC1 variability. Females with higher anxiety showed reduced variability in pitch slope. Correlations did not survive multiple testing correction.

**Conclusions:**

Acoustic speech markers elicited in socially evaluative contexts can accurately recognize SAD in male adolescents three years in advance. Performance is moderate for females and other anxiety disorders, underscoring the need for sex-specific approaches to diagnostic tool development.

## Background

Anxiety disorders are the most prevalent mental health disorders in adolescents and are associated with adverse psychosocial outcomes (Rockhill et al., 2010). They often manifest early, with a mean age of onset of 11 years (Kessler et al., 2005), yet they remain largely undertreated. When left untreated, anxiety disorders often become chronic and predict other mental health conditions in adulthood (Essau, Conradt, & Petermann, 2002). One critical factor in the underrecognition of anxiety disorders in young people is the perception that anxiety symptoms are intrinsic personality traits rather than treatable conditions (Wang, Berglund, Olfson, & Kessler, 2004). Additionally, many adolescents are reluctant to discuss their anxiety symptoms and seek help due to stigma concerns (Gulliver, Griffiths, & Christensen, 2010). This is unfortunate, as effective, non-stigmatizing treatments for anxiety disorders are available (Siegel & Dickstein, 2011). A low-intensity, low-cost screening method for detecting anxiety disorders that does not require young people to verbalize their symptoms could be a first step in the early detection of anxiety disorders, helping those who need professional treatment and thereby preventing the stagnation of their social and educational development.

One potential non-confrontational screening method for anxiety disorders is through the automatic extraction of acoustic-prosodic markers from speech. Despite their heterogeneity, anxiety disorders consistently exhibit a strong physical component. Anxiety increases muscle tension and respiratory rate (Horvath & Fenz, 1971), altering the mechanics of speech production and, consequently, the acoustic-prosodic properties of speech sounds (Sondhi et al., 2015).

Several studies have identified cross-sectional associations between anxiety and speech characteristics in adults. Anxiety often affects the fundamental frequency (F0) of speech, which is the rate at which the vocal cords vibrate (open and close) across the glottis during phonation, perceived as pitch. Most studies have found that mean F0 increases with anxiety (Hagenaars & van Minnen, 2005; Laukka et al., 2008; Teferra et al., 2022; Weeks et al., 2012), although others report no change (Goberman, Hughes, & Haydock, 2011; Wortwein, Morency, & Scherer, 2015). Anxiety-induced muscle tension can also alter formant frequencies (F1, F2, F3), which define vowel sounds by reflecting the concentration of acoustic energy at specific frequencies of the vocal tract (Özseven, Düğenci, Doruk, & Kahraman, 2018; Wortwein et al., 2015). Temporal aspects of speech, such as speech rate and pause frequency, may also be influenced by anxiety. Some studies indicate that anxious individuals speak faster and pause less (Murray & Arnott, 1993; Siegman & Boyle, 1993), while others suggest the opposite, with higher anxiety associated with slower speech and more frequent pauses (Goberman et al., 2011; Hagenaars & van Minnen, 2005; Laukka et al., 2008; Teferra et al., 2022;). Anxiety has also been associated with voice quality as assessed by features such as jitter (cycle-to-cycle F0 variation), shimmer (cycle-to-cycle loudness variation), and Mel-Frequency Cepstral Coefficients (MFCC) (spectral properties of speech sounds), but findings vary across studies (Jones, Anagnostou, & Verhoeven, 2011; Özseven et al., 2018; Teferra et al., 2022). All speech deviations in anxious individuals may be exacerbated under stress, such as during public speaking situations.

So far, no study has investigated this relationship among adolescents, a critical period for anxiety onset. Adolescent speech differs from that of adults due to rapid developmental changes affecting their voice, including the widening of the larynx and elongation of the vocal tract (Zamponi, Mazzilli, Mazzilli, & Fantini, 2021). Compared to adults, they exhibit higher pitch and formant frequencies, longer durations of individual phonetic segments, and greater variability in temporal and spectral features (Lee, Potamianos, & Narayanan, 1999). Thus, anxiety may manifest differently in adolescent speech, potentially necessitating specific acoustic markers for accurate detection.

Furthermore, voice changes in adolescents follow different developmental trajectories in males and females, with males experiencing more pronounced transformations (Zamponi et al., 2021). Male adolescents experience substantial decreases in F0 and formant frequencies, resulting in lower-pitched, more resonant voices compared to females (Markova et al., 2016).

The above-described findings show that, although several studies have assessed associations between acoustic-prosodic markers and anxiety in adults, the direction of effects has remained inconclusive, with most studies relying on cross-sectional analyses. Assessing the predictive potential of speech quantification in relation to the risk of developing anxiety in the future is needed for understanding its value in screening and early detection.

In the present study, we evaluated cross-sectional and longitudinal associations between anxiety and acoustic-prosodic markers, using data from a longitudinal cohort of Dutch adolescents collected during a standardized social stress test. First, we conducted an exploratory, sex-specific analysis of the associations between speech patterns and anxiety symptoms in adolescents around age 16. Subsequently, we evaluated the potential of using sex-specific acoustic markers assessed at age 16 to predict the severity of anxiety symptoms and the diagnosis of an anxiety disorder at age 19. Given the nature of the speech elicitation task – a test designed to induce stress in socially evaluative situations – we hypothesize that this task will be particularly effective at eliciting speech markers specific to social anxiety disorder (SAD), thereby yielding greater accuracy in detecting SAD than a composite anxiety diagnosis including various anxiety disorders. Additionally, drawing on previous research (Weeks et al., 2012), we hypothesize that SAD will be more easily recognized through acoustic speech markers in male than in female adolescents.

## Methods

### Participants

This study is based on data from a subsample of 581 participants (51.1% females) selected from the longitudinal population-based TRacking Adolescents’ Individual Lives Survey (TRAILS) (Oldehinkel et al., 2015), which includes biennial or triennial assessments from age 11 onwards. The total sample comprised 2230 participants, of whom 715 agreed to participate in a series of additional tests, including a digitally recorded speech task (Oldehinkel et al., 2011). Of these speech recordings, 581 were of sufficient quality for quantitative analysis (see subsection 2.3 below). The data were collected when participants were aged 15–17 years (mean = 15.7, SD = 0.67) and 18–20 years (mean = 18.5, SD = 0.59) respectively. Inclusion criteria required consent of the child’s primary school to participate in the study. Children were excluded if they had a severe intellectual disability, a significant physical illness or impairment, or if no Dutch-speaking parent or guardian was available to assist with study procedures. For a full description of the inclusion and exclusion criteria, see de Winter et al. (2005). Written informed consent was obtained from both parents and adolescents prior to enrollment. The study was approved by the Dutch Central Committee on Research Involving Human Subjects (CCMO) and adhered to the principles of the Declaration of Helsinki.

### Speech elicitation task

At baseline, participants completed the speech task from the Groningen version of the ‘Trier Social Stress Test’ (GTSST), a protocol originally designed to create a moderate level of performance-related psychological stress in a lab environment using various tasks (Kirschbaum, Pirke, & Hellhammer, 2008). For the GTSST, participants were instructed in loco to prepare and deliver a monologue, in which they had to speak continuously for 6 minutes about themselves and their lives. Participants delivered the monologue in front of both a video camera and a research assistant. Prior to the task, they were informed that their recorded performance would later be evaluated and ranked by a panel of peers based on speech content, use of voice, and posture. During the task, the research assistant observed the participant with a critical disposition. The speech task took place in test rooms located near participants’ residences (e.g., hotels, community centers). All rooms were soundproof with blinded windows. Built-in microphones integrated with video cameras were consistently used for recording. The experimental setup (i.e., position of the camera in relation to the participant) was similar in all sessions.

### Acoustic analyses

Each participant’s audio channel was extracted from the video recording using the FFmpeg library (https://ffmpeg.org/). Audio quality was assessed by a trained researcher who manually inspected spectrograms using Praat (Boersma & Weenink, 2013), checking for clipping, background noise, and human intelligibility.

The 581 audio recordings were acoustically analyzed with OpenSMILE (version 2.0), employing the extended Geneva Acoustic Minimalistic Parameter Set (eGeMAPS) (Eyben, Weninger, Gross, & Schuller, 2013). This set contains 88 acoustic parameters, sorted into four categories: frequency parameters (28 parameters, e.g., fundamental frequency), energy/amplitude parameters (10 parameters, e.g., loudness), spectral balance parameters (44 parameters, e.g., formants), and temporal parameters (6 parameters, e.g., speech rate). Parameters were derived from 18 low-level descriptors, for which the arithmetic mean and coefficient of variation were calculated. For pitch and loudness, eight additional measures were computed: 20-th, 50-th, and 80-th percentiles, percentile range (20-th to 80-th), and mean and standard deviation of falling and rising slope. These additional measures, part of the eGeMAPS set (Eyben et al., 2016), capture both distributional (percentiles) and dynamic (slopes) aspects of pitch and loudness.

### Anxiety measures

Severity of anxiety symptoms was evaluated at baseline using the Youth Self Report ([YSR]; Achenbach & Rescorla, 2001) and at follow-up using the Adult Self Report ([ASR]; Achenbach & Rescorla, 2003). The YSR includes six items and the ASR includes seven items related to anxiety symptoms. For both the YSR and ASR, the anxiety scale scores were calculated by averaging the anxiety-related item scores. The YSR demonstrated moderate internal consistency with a Cronbach’s alpha of 0.65, while the ASR showed good internal consistency with a Cronbach’s alpha of 0.75.

At follow-up, the World Health Organization Composite International Diagnostic Interview (CIDI 3.0) was used to assess the presence of anxiety disorders according to the Diagnostic and Statistical Manual of Mental Disorders (DSM-IV) (Haro et al., 2006). Diagnoses included lifetime occurrences of adult separation anxiety, agoraphobia, generalized anxiety disorder, panic disorder, childhood separation anxiety, social anxiety, and specific phobia. Individuals diagnosed with an obsessive-compulsive disorder were excluded from this study as the DSM-5 reclassifies this condition outside the category of anxiety disorders (2013) (American Psychiatric Association, 2013). A composite anxiety disorder measure was created based on the presence of any of the seven specified diagnoses, with participants having at least one of these disorders being classified as having an anxiety disorder (hereafter referred to as AD).

### Statistical analyses

#### Data transformation

Boxplots based on the interquartile range (IQR) were used to visually inspect the distributions of acoustic features. To quantify the asymmetry of each distribution, the skewness coefficient was calculated for each feature. Twenty-four acoustic variables with skewed distributions (skewness coefficient above +1 or below −1) were transformed to better fit a normal distribution (Supplementary Table S1). A logarithmic transformation was applied to variables with mild positive skewness (skewness coefficient between +1 and + 3), and a reciprocal transformation (1/x) was applied to those with severe positive skewness (skewness coefficient greater than +3). For negatively skewed variables (skewness coefficient less than −1), square transformation was applied. Variables that remained skewed (skewness coefficient greater than +1 or less than −1) after transformation were excluded, resulting in the removal of five variables, leaving 83 parameters for analysis (for a list, see Supplementary Table S2). All statistical analyses were performed with R (version 4.1.2) (R Core Team, 2020).

#### Cross-sectional associations with anxiety symptoms

Independent two-tailed bivariate Spearman’s correlations were calculated between each of the 83 speech features and baseline anxiety severity to investigate cross-sectional relationships. The Benjamini-Hochberg (BH) method was employed to adjust for the high false discovery rate (FDR) due to multiple correlation tests. Analyses were performed separately for males and females.

#### Longitudinal associations with anxiety symptoms

Backward linear regression models were used to test the longitudinal relationship between speech features and anxiety severity at follow-up. To avoid multicollinearity, pairs of highly intercorrelated acoustic features (i.e., Spearman’s rho > .85) were identified (Elith et al., 2006), and the feature with the highest average correlation to other features was excluded (Supplementary Table S3). The backward elimination process, based on the Akaike information criterion (AIC), systematically removed one predictor at a time until further removals lowered the AIC. Standardized regression coefficients (Beta) were used to rank predictors by their relative importance.

To assess the predictive effect of speech features on follow-up anxiety beyond baseline anxiety, a linear regression model was first constructed using baseline anxiety as the sole predictor. In a second model, acoustic features were added to baseline anxiety. The fits of the two models were compared by means of *F*-statistics. Analyses were performed separately for male and female participants.

#### Longitudinal classification of anxiety disorders

Binary Random Forest (RF) classifiers (Breiman, 2001) were used to distinguish individuals with a lifetime anxiety disorder (AD) and a social anxiety disorder (SAD) from healthy controls based on speech features. To create balanced groups, the dataset was down sampled to include 246 participants for AD (123 with AD, 123 without) and 108 for SAD (54 with SAD, 54 without), matched for sex, age, and education. All speech features were standardized to have a mean of 0 and a standard deviation of 1 before being used in the model. All models were trained using 10-fold cross-validation, where in each iteration, the model was trained on data from 9 folds and tested on the remaining fold. This process was repeated 10 times, ensuring that each fold served as a test set once. Classification was performed using a subset of features selected through Recursive Feature Elimination with Cross-Validation (RFECV). RFECV iteratively removed the least informative features one at a time based on accuracy, using 10-fold cross-validation. Gini importance scores were computed to compare the relative ranking of acoustic features in the classifiers. Model performance was evaluated using accuracy, sensitivity, specificity, and the area under the receiver operating characteristic curve (AUC-ROC). All analyses were conducted separately for males and females.

## Results

### Descriptive statistics

Table 1 presents demographic and clinical information of the participants, stratified by sex. Female participants exhibited higher levels of anxiety at both baseline and follow-up compared to males (Table 1). A total of 123 participants met criteria for at least one anxiety disorder (AD)(Supplementary Table S4). This group included 54 individuals diagnosed with social anxiety disorder (SAD). Of those with SAD, 21 also met criteria for at least one additional AD. Educational level did not differ between males and females (Table 1), nor between anxious and non-anxious participants (Supplementary Table S4).

**Table 1.** Demographic and clinical characteristics of participants stratified by sex

	Full sample (*n* = 581)			
	Males (*n* = 284)	Females (*n* = 297)	Statistics	*p* Value
Age at baseline (y)	15.7 ± 0.69	15.6 ± 0.65	*T* = −0.507	*p* = .612
Age at follow-up (y)	18.5 ± 0.60	18.5 ± 0.57	*T* = −1.092	*p* = .275
Education level at baseline				
Lower vocational and special education	67 (26%)	62 (22%)	*Χ^2^* = 0.512	*p* = .474
Intermediate vocational	71 (27%)	77 (27%)	*Χ^2^* = 0.039	*p* = .843
Higher vocational	63 (24%)	58 (21%)	*Χ^2^* = 0.520	*p* = .471
Academic	60 (23%)	83 (30%)	*Χ^2^* = 2.671	*p* = .102
Anxiety severity				
YSR at baseline	0.2 ± 0.25	0.4 ± 0.32	*T* = 7.296	*p* < .001
ASR at follow-up	0.3 ± 0.30	0.4 ± 0.36	*T* = 5.239	*p* < .001
DSM IV anxiety diagnosis at follow-up^a^				
No disorder	242 (85%)	216 (73%)		
Adult separation anxiety	3 (1%)	10 (3%)		
Agoraphobia	0 (0%)	5 (2%)		
Generalized anxiety disorder	4 (1%)	15 (5%)		
Panic disorder	0 (0%)	2 (1%)		
Childhood separation anxiety	7 (2%)	12 (4%)		
Social anxiety disorder	22 (8%)	32 (11%)		
Specific phobia	17 (6%)	40 (13%)		

*Note:* Reported values are means ± SD or n (%).

Abbreviations: ASR, adult self report; *N*, sample size; y, years; YSR, youth self report.

a Percentages exceed 100% because some individuals were diagnosed with multiple anxiety disorders.

### Cross-sectional associations with anxiety symptoms

Significant associations were identified between baseline anxiety and four acoustic-prosodic features in males and one in females (Supplementary Figure S1). However, none of these correlations remained statistically significant after false discovery rate correction for multiple comparisons (based on 83 tests). In males, three of the associated features pertained to pitch and formants, while one was related to voice quality. Higher anxiety severity was negatively correlated with the mean pitch falling slope (rho = −0.166, *p* = .006), pitch range (20th to 80th percentile) (rho = −0.189, *p* = .002), and mean frequency of the first formant (F1) (rho = −0.127, *p* = .036), indicating that increased anxiety is associated with a slower pitch falling rate, a narrower pitch range, and a lower F1 frequency. Conversely, anxiety was positively correlated with the coefficient of variation of MFCC1 (rho = 0.132, *p* = .029). In females, a negative association was observed between the standard deviation of the pitch falling slope and anxiety (rho = −0.119, *p* = .043), indicating that a lower variation in the rate at which pitch falls is linked to higher anxiety severity (Supplementary Figure S1).

### Longitudinal associations with anxiety symptoms

After removing highly intercorrelated features (i.e., Spearman’s rho > .85), 55 speech parameters were analyzed using backward linear regression models to predict anxiety levels at 3-year follow up, separately for males and females. In male participants, the optimal model identified nine features, accounting for 24.6% of the variance (Table 2). Adding speech features resulted in a 5.4% increase in explained variance compared to a model with only baseline anxiety. This increase was statistically significant (F(8, 238) = 2.567, *p* = .002). For females, the best-fitting regression model included 23 predictors which explained 33.2% of the variance in future anxiety (Table 2). Adding speech features to a model that already accounted for baseline anxiety resulted in a 10.9% increase in explained variance. This improvement was statistically significant (*F*(22, 253) = 3.026, *p* < .001). The top three predictors of future anxiety severity for males and females are presented in Table 3 (for a full list, see Supplementary Table S5).

**Table 2.** Linear regression results predicting anxiety severity at 3-year follow-up using acoustic-prosodic features, baseline anxiety, and a combined model including both

Males	Feature (*n*)	Adjusted *R* ^2^	RSE	*F* statistic	*p* Value
Acoustic features	15	*R* ^2^ = 0.121	0.282	*F*(15,232) = 3.270	.041
Baseline anxiety severity	1	*R* ^2^ = 0.192	0.272	*F*(1,246) = 59.200	.001
**Baseline anxiety severity + acoustic features**	**9**	***R*** ^**2**^ **= 0.246**	**0.259**	***F*(9,238) = 9.960**	**.001**
Females					
Acoustic features	16	*R* ^2^ = 0.134	0.336	*F*(16, 260) = 3.670	.011
Baseline anxiety severity	1	*R* ^2^ = 0.223	0.319	*F*(1, 273) = 79.690	.001
**Baseline anxiety severity + acoustic features**	**23**	***R*** ^**2**^ **= 0.332**	**0.295**	***F*(23, 253) = 6.967**	**.001**

*Note:* Best performing model (highest *R* ^2^, lowest RSE) in bold.

Abbreviations: *N*, number; RSE, residual standard error.

**Table 3.** Top three variables predicting anxiety severity at 3-year follow-up in male and female adolescents, ranked by absolute value of standardized coefficients (highest to lowest)

Males	*β*	*b*	SE	*p* Value
Baseline anxiety severity	0.399	0.510	0.072	<.001
Loudness falling slope (mean)	−0.357	−0.089	0.026	<.001
Loudness peaks (per second)	0.340	0.158	0.039	<.001
**Females**				
logRel F0-H1-A3 (mean)	0.613	0.077	0.017	<.001
Baseline anxiety severity	0.448	0.501	0.057	<.001
Voiced segments (per second)	0.442	0.256	0.057	<.001

Abbreviations: *β*, standardized coefficient; *b*, unstandardized coefficient; SE, standard error.

### Longitudinal classification of anxiety disorders

**Figure 1.**
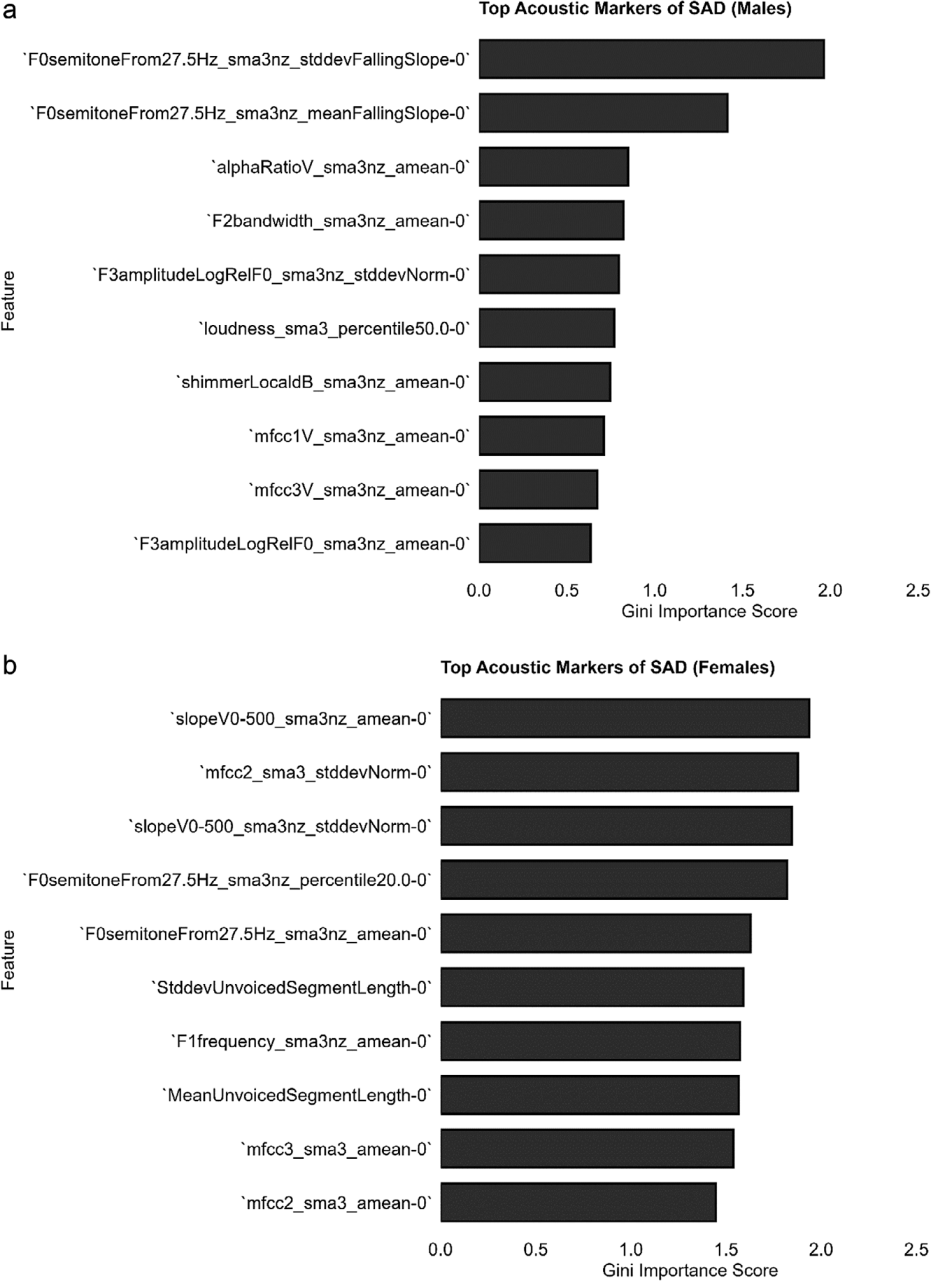
Top 10 acoustic-prosodic features ranked by Gini importance for the longitudinal classification of Social Anxiety Disorder (SAD) in males (a) and females (b). Feature importance was computed as the total decrease in Gini impurity per feature across all trees in the random forest classifier.

The demographic characteristics of the matched subset of 246 participants, used for the random forest classifiers, are presented in Supplementary Table S4. The RF model achieved the highest performance in distinguishing individuals with a lifetime diagnosis of SAD at age 19, with an AUC-ROC of 85% for males (*N* = 44) and 74% for females (*N* = 64) (Table 4). The accuracy was 74% for males and 68% for females. The top 10 discriminative features for SAD were primarily related to pitch and voice quality in males, whereas in females, they were associated with voice quality, pitch, and pauses (Figure 1). Additionally, the RF model achieved an AUC–ROC of 73% for males (*N* = 84) and 67% for females (*N* = 162) when classifying adolescents with a lifetime diagnosis of any anxiety disorder (AD) at age 19 (Table 4). The accuracy was 68% for males and 61% for females. The complete list of features selected for SAD and AD classification can be found in the supplementary materials (Supplementary Table S6).

**Table 4.** Classification performance of random forest models predicting anxiety (AD) and social anxiety disorder (SAD) diagnoses at 3-year follow-up in male and female adolescents

Males	Diagnosis	Feature (*n*)	AUC–ROC	Accuracy	Sensitivity	Specificity
	Anxiety diagnosis (AD)	23	73.4%	67.9%	69.0%	66.7%
	**Social anxiety disorder (SAD)**	**25**	**85.4%**	**73.5%**	**74.2%**	**72.7%**
Females						
	Anxiety diagnosis (AD)	30	66.9%	60.8%	69.2%	52.7%
	**Social anxiety disorder (SAD)**	**27**	**73.9%**	**67.7%**	**69.7%**	**65.6%**

*Note:* Best performing model in bold.

Abbreviations: *N*, number; AUC–ROC, area under the curve–receiver operating characteristic.

## Discussion

This is the first study to assess cross-sectional and longitudinal associations between acoustic-prosodic markers and anxiety in a large sample of male and female adolescents representative of the general (Dutch) population. The model accurately identified SAD three years in advance in males, achieving an AUC–ROC of 85%, and demonstrated moderate accuracy in females, with an AUC–ROC of 74%. Speech features improved the clinical prediction of anxiety severity at three years, with distinct acoustic predictors identified for males and females in the linear regression models.

### Cross-sectional associations with anxiety symptoms

Several acoustic features were cross-sectionally correlated with anxiety severity; however, none remained statistically significant after correction for multiple testing and they should thus be interpreted with caution. In both sexes, anxiety severity was negatively associated with the falling slope of F0 (pitch). Pitch contours, characterized by rising and falling slopes, contribute to intonation and convey information about linguistic meaning, syntactic structures, and speaker emotions (Laukka, 2017; Vaissiére, 2008). Females with higher anxiety exhibited a falling pitch trajectory with reduced variability, while anxious males showed a slower pitch falling rate. Both results suggest reduced pitch dynamism, consistent with emotion regulation theories proposing that anxious individuals suppress their emotional expressiveness, particularly in socially evaluative situations like the Trier Social Stress Test used in this study (Gross, 2002).

In males, higher anxiety was associated with a narrower pitch range, indicating a reduced span of vocal frequencies in their speech. This aligns with previous research by Drioli, Tisato, Cosi, and Tesser (2003) which demonstrated a lower pitch range in a male university student during automatic fear detection compared to other emotions. No such effect emerged in females, potentially due to their naturally wider pitch range (195–275 Hz in females vs. 140–214 Hz in males; Wilson, 1987), which may make anxiety-related increases harder to detect.

The frequency of the first formant (F1) was negatively correlated with anxiety in males. F1 reflects the physical characteristics of the vocal tract, and is negatively correlated with tongue height during speech (Lee, Shaiman, & Weismer, 2016). Anxiety-induced tension in the supraglottal articulators can alter their movement and positions, influencing F1 values. This partly aligns with previous research showing reduced F1 in anxious states, although without sex-specific effects (Özseven et al., 2018). Another study observed a correlation between anxiety and F1 during a social stress test in adult males but not in adult females (Teferra et al., 2022), just like we did, but in the opposite direction. This discrepancy may reflect age differences between the study samples (mean ages of approximately 16 and 36). During adolescence, anatomical changes in the male vocal tract lower F1 frequency, with maturation continuing until around age 19 (Lee et al., 1999). These changes may modulate the anxiety–F1 relationship in adolescents. Lastly, MFCC1 variability positively correlated with anxiety in males, suggesting more fluctuations in energy distribution across frequencies, likely due to articulatory instability from supraglottal muscle tension. Given that the task was self-referential and likely to elicit social-evaluative concerns – a central feature of social anxiety – future studies should investigate associations between state social anxiety during the TSST and acoustic markers, to better disentangle these effects from those of trait anxiety.

### Longitudinal associations with anxiety symptoms

Our longitudinal analyses demonstrated that acoustic features predicted anxiety severity at age 19, after accounting for baseline anxiety at age 16. The additional variance explained by acoustic features (5.4% for males, 10.9% for females) demonstrates that speech markers provide incremental predictive value beyond baseline anxiety levels. This suggests that these acoustic features capture information not redundant with traditional self-reported measures of anxiety, enhancing predictive precision.

Acoustic features predictive of future anxiety severity varied by sex, confirming our initial hypothesis that accurate prediction of anxiety disorders may benefit from developing sex-specific prediction models. Loudness emerged as the strongest acoustic predictor of future anxiety in male adolescents. Males with higher anxiety at age 19 exhibited more loudness peaks in their speech and a slower rate of loudness decline at age 16. Changes in loudness are one of the main characteristics contributing to the perception of instability in the voice of male adolescents (Boltez̆ar, Burger, & Z̆argi, 1997), often attributed to the growth of their respiratory system and the necessary adaptation of nervous control over the speech apparatus. Increased loudness has also been linked to heightened emotional arousal (Siegman & Boyle, 1993). Together, these results suggest that a stressful situation may exacerbate loudness instabilities in individuals vulnerable to developing anxiety.

Females with higher levels of anxiety at age 19 spoke faster with more alterations in vocal quality. This aligns with studies linking anxiety to a faster speech rate (Murray & Arnott, 1993; Siegman & Boyle, 1993) and highlights its potential as an early marker of anxiety in female adolescents. Women generally experience increased arousal and quicker responses to stress compared to men (Bangasser, Eck, Telenson, & Salvatore, 2018), which may help explain their faster speech rate in such situations. In females predisposed to anxiety, this response may become maladaptive and predict greater future anxiety severity (Dieleman et al., 2016; Nelemans et al., 2017). Overall, these findings suggest that speech patterns not only reflect current anxiety but may also serve as early markers of increased vulnerability to developing anxiety over time.

### Longitudinal classification of anxiety disorders

The RF model trained on selected feature subsets achieved the highest AUC–ROC for the longitudinal classification of individuals with a lifetime diagnosis of SAD: 85% for males and 74% for females. Notably, these findings were derived from a non-clinical, non-help-seeking population, reinforcing the potential of speech markers for the early detection of SAD in the general population. Despite females being more likely to receive a diagnosis of SAD and reporting more severe symptoms than males, the speech-based automatic classification model had greater accuracy in males. The most informative feature for classifying SAD in males was the falling slope of pitch, which we also found to correlate cross-sectionally with anxiety severity. These findings align with prior research showing that pitch during social threat tasks can serve as a physiological marker of SAD in males, with effects that do not always generalize or are weaker in females (Weeks et al., 2012). From a developmental perspective, Scharfstein et al. (2011) found that children with SAD had higher pitch during a role play assessment than children with Asperger’s disorder, though neither differed significantly from typically developing peers. Our results support and extend these findings by showing that, during adolescence, the association between pitch and SAD relative to healthy groups becomes more pronounced in males, with a more modest effect observed in females, as indicated by the selected features in the RF model. Future studies could examine whether pubertal voice maturation helps explain why pitch-based features are more predictive of SAD in boys than in girls. In males, puberty is accompanied by a substantial drop in pitch, and delays in this process have been linked to increased peer-related stress and social withdrawal (Dwyer, 2020), which may increase vulnerability to social anxiety. We recommend that future research include measures of vocal maturation to test this hypothesis.

As hypothesized, the SAD classification model outperformed the overall anxiety model (AUC-ROC of 72.4% for males and 63.3% for females). This finding may be attributed to the heterogeneity of anxiety disorders, which involve diverse symptom profiles and triggers (Drzewiecki & Fox, 2024). While social anxiety is primarily triggered by fear of social evaluations, other anxiety disorders may be influenced by different stimuli, such as specific situations or sensory challenges, which were not elicited in this study. The superior performance of the SAD model may be due to the fact that the Trier Social Stress Test is designed to elicit stress responses that may particularly trigger individuals with SAD, in turn eliciting speech responses that directly tap into this disorder. Consistent with this, prior research has shown that the association between pitch and SAD in a socially evaluative task was not attributable to general arousal or symptoms of generalized anxiety or panic disorder but was specific to SAD (Weeks et al., 2012). These findings underscore the critical role of task design in detecting speech markers for anxiety disorders.

### Limitations

This study has some limitations that warrant consideration. First, dividing the sample by sex reduced the statistical power, particularly in the analysis of anxiety subtypes, highlighting the need for replication in larger samples to validate the findings. Second, after normalizing the correlation results across 83 tests, none of the correlation analyses reached statistical significance. This outcome was anticipated given the high number of features examined, which increased the likelihood of non-significant results after correction for multiple testing. Future studies may benefit from feature reduction techniques to mitigate this challenge. A third limitation is that, although groups did not differ in educational level – a proxy for cognitive ability (Huh et al., 2024) – we did not assess whether cognitive functioning moderates the relationship between speech features and anxiety, which could be explored in future research. Similarly, future research may account for comorbid conditions such as Autism Spectrum Disorder (ASD) or depression, which may influence both speech production and anxiety levels.

Last, we used lifetime anxiety disorders that could have started before the assessment of speech, as power restrictions did not allow focusing on onsets after the speech assessment only. However, we did adjust for pre-assessment anxiety in the analyses with symptom severity as outcome measure and these analyses still indicated a prospective effect. Future studies may prospectively follow adolescents without a baseline diagnosis and collect repeated speech samples alongside clinical assessments to assess whether acoustic features can predict the onset of anxiety disorders.

### Conclusion

The current study demonstrates that acoustic speech markers elicited in a socially evaluative context are most predictive for the early detection of SAD in male adolescents, with modest accuracy in females and in other anxiety disorders. Speech analyses may thus aid in recognizing vulnerable adolescents at risk of developing anxiety disorders, facilitating early intervention.

## Supporting information

Ciampelli et al. supplementary material Ciampelli et al. supplementary material

## Supplementary material

The supplementary material for this article can be found at http://doi.org/10.1017/S0033291725102274.
