# Supplementary material for: Cross-sectional and longitudinal associations between anxiety and acoustic-prosodic markers in adolescents
Source: Psychol Med. 2025 Oct 28;55:e322. doi: 10.1017/S0033291725102274 (PMC13054901; doi:10.1017/S0033291725102274)
Supplement: Ciampelli et al. supplementary material [file S0033291725102274sup001.docx]

| ***Table S1.*** | | |
| --- | --- | --- |
| List of acoustic features subject to data transformation. | | |
|  | **Acoustic feature** | **Transformation method** |
| 1 | F0semitoneFrom27.5Hz_sma3nz_stddevNorm-0 | logarithmic transformation |
| 2 | F0semitoneFrom27.5Hz_sma3nz_pctlrange0-2-0 | logarithmic transformation |
| 3 | loudness_sma3_meanRisingSlope-0 | logarithmic transformation |
| 4 | loudness_sma3_stddevRisingSlope-0 | logarithmic transformation |
| 5 | loudness_sma3_stddevFallingSlope-0 | logarithmic transformation |
| 6 | spectralFlux_sma3_amean-0 | logarithmic transformation |
| 7 | spectralFlux_sma3_stddevNorm-0 | logarithmic transformation |
| 8 | mfcc2_sma3nz_stddevNorm-0 | logarithmic transformation |
| 9 | mfcc3_sma3nz_stddevNorm-0 | reciprocal transformation |
| 10 | mfcc4_sma3nz_stddevNorm-0 | reciprocal transformation |
| 11 | HNRdBACF_sma3nz_stddevNorm-0 | square transformation |
| 12 | logRelF0-H1-H2_sma3nz_stddevNorm-0 | reciprocal transformation |
| 13 | logRelF0-H1-A3_sma3nz_stddevNorm-0 | logarithmic transformation |
| 14 | jitterLocal_sma3nz_amean-0 | logarithmic transformation |
| 15 | slopeV0-500_sma3nz_stddevNorm-0 | logarithmic transformation |
| 16 | slopeV500-1500_sma3nz_stddevNorm-0 | square transformation |
| 17 | spectralFluxV_sma3nz_amean-0 | logarithmic transformation |
| 18 | spectralFluxV_sma3nz_stddevNorm-0 | logarithmic transformation |
| 19 | mfcc2V_sma3nz_stddevNorm-0 | square transformation |
| 20 | mfcc3V_sma3nz_stddevNorm-0 | reciprocal transformation |
| 21 | mfcc4V_sma3nz_stddevNorm-0 | reciprocal transformation |
| 22 | spectralFluxUV_sma3nz_amean-0 | logarithmic transformation |
| 23 | MeanUnvoicedSegmentLength-0 | logarithmic transformation |
| 24 | StddevUnvoicedSegmentLength-0 | logarithmic transformation |

| ***Table S2.*** | |
| --- | --- |
| List of acoustic features discarded after data transformation. | |
|  | **Acoustic feature** |
| 1 | HNRdBACF_sma3nz_stddevNorm-0 |
| 2 | slopeV500-1500_sma3nz_stddevNorm-0 |
| 3 | mfcc2V_sma3nz_stddevNorm-0 |
| 4 | mfcc3V_sma3nz_stddevNorm-0 |
| 5 | mfcc4V_sma3nz_stddevNorm-0 |

| ***Table S3.*** | |
| --- | --- |
| List of acoustic features excluded from linear regressions due to high inter-correlation (i.e., Spearman’s rho > .85). | |
|  | **Acoustic feature** |
| 1 | F0semitoneFrom27.5Hz_sma3nz_percentile20.0-0 |
| 2 | F0semitoneFrom27.5Hz_sma3nz_amean-0 |
| 3 | loudness_sma3_amean-0 |
| 4 | loudness_sma3_percentile80.0-0 |
| 5 | loudness_sma3_percentile50.0-0 |
| 6 | loudness_sma3_meanRisingSlope-0 |
| 7 | spectralFlux_sma3_amean-0 |
| 8 | spectralFluxV_sma3nz_amean-0 |
| 9 | mfcc1_sma3_amean-0 |
| 10 | mfcc2_sma3_stddevNorm-0 |
| 11 | mfcc2_sma3_amean-0 |
| 12 | mfcc3_sma3_stddevNorm-0 |
| 13 | mfcc3_sma3_amean-0 |
| 14 | mfcc4V_sma3nz_amean-0 |
| 15 | mfcc4_sma3_amean-0 |
| 16 | HNRdBACF_sma3nz_amean-0 |
| 17 | logRelF0-H1-H2_sma3nz_stddevNorm-0 |
| 18 | F1frequency_sma3nz_amean-0 |
| 19 | F1amplitudeLogRelF0_sma3nz_stddevNorm-0 |
| 20 | F1amplitudeLogRelF0_sma3nz_amean-0 |
| 21 | F2frequency_sma3nz_amean-0 |
| 22 | F2amplitudeLogRelF0_sma3nz_stddevNorm-0 |
| 23 | F2amplitudeLogRelF0_sma3nz_amean-0 |
| 24 | F3amplitudeLogRelF0_sma3nz_stddevNorm-0 |
| 25 | alphaRatioV_sma3nz_amean-0 |
| 26 | alphaRatioV_sma3nz_stddevNorm-0 |
| 27 | slopeV0-500_sma3nz_amean-0 |
| 28 | hammarbergIndexUV_sma3nz_amean-0 |

|  |
| --- |


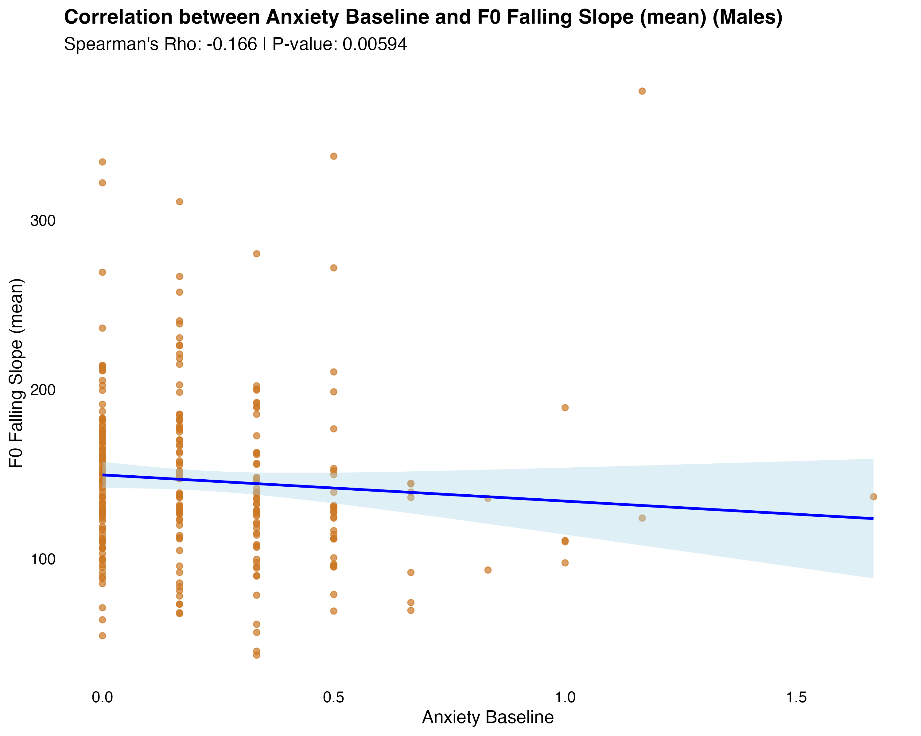


Figure S1. Scatterplots showing the correlations between acoustic-prosodic features and baseline anxiety. The blue line represents the fitted linear regression model, and the shaded area around the line indicates the 95% confidence interval for the regression line. Spearman's correlation coefficients and p-values are displayed in the subtitle.


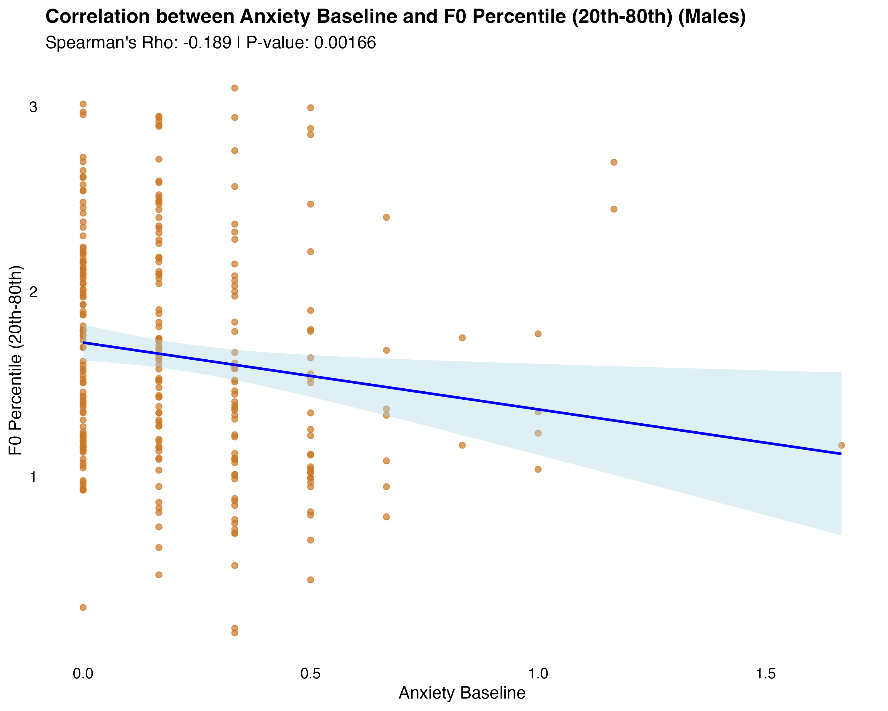


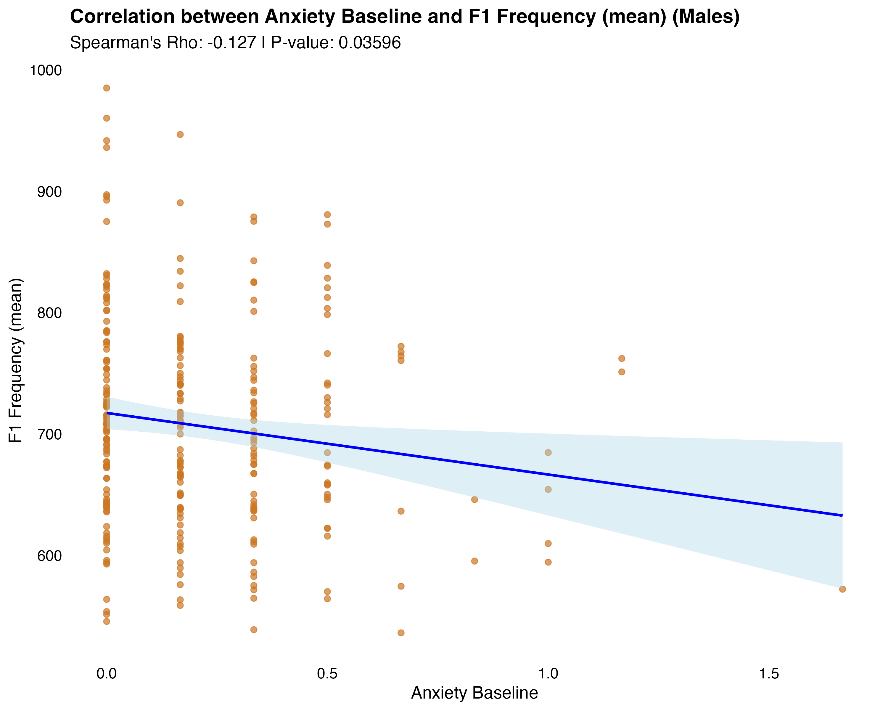


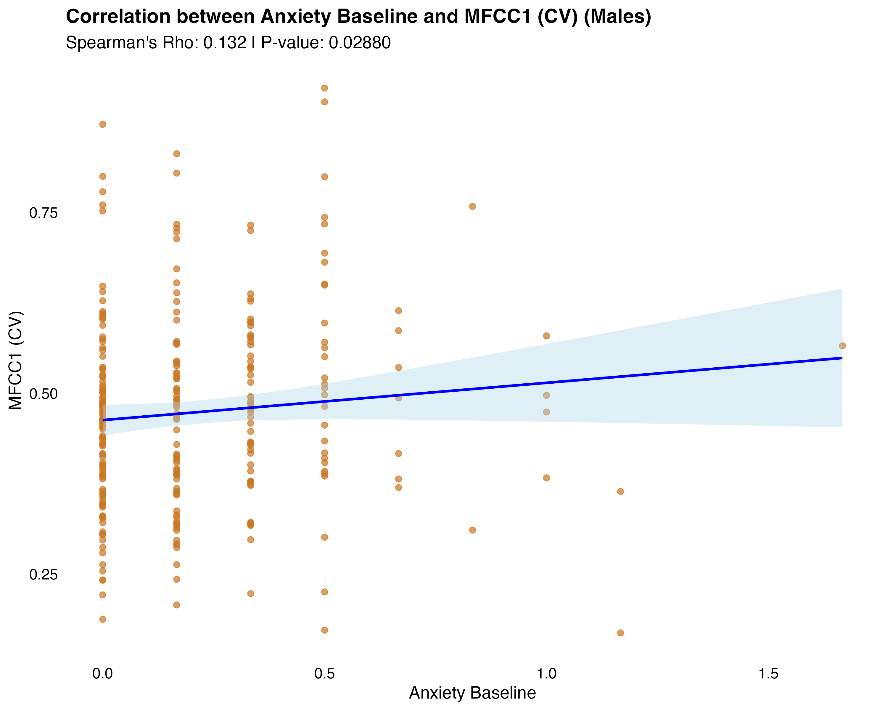


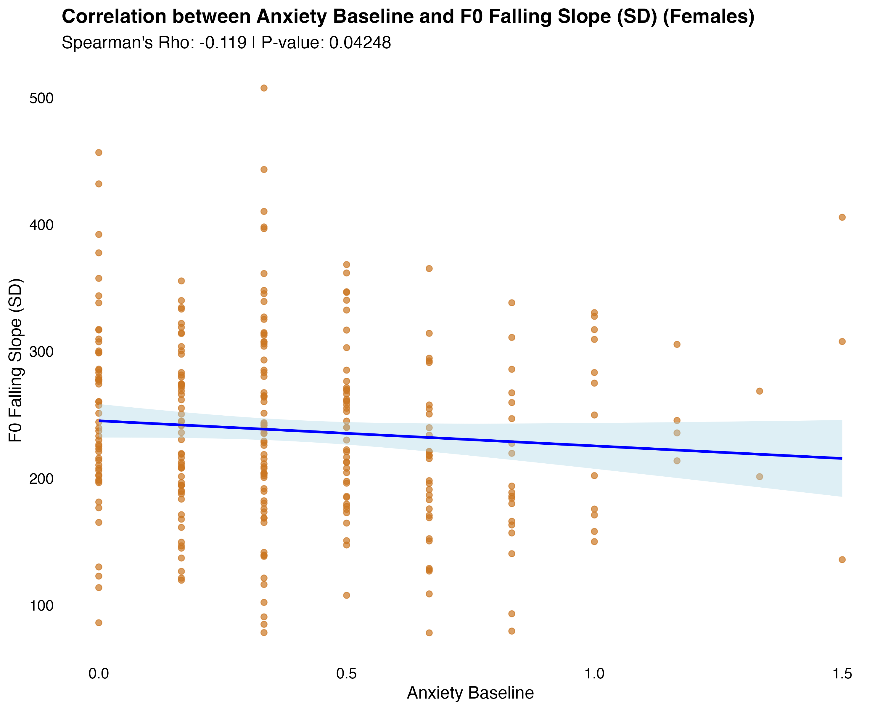


|  | ***Table S4.*** | | | | | | | |  |
| --- | --- | --- | --- | --- | --- | --- | --- | --- | --- |
|  | Demographic characteristics of male and female participants that met the criteria for an anxiety disorder at follow-up and healthy participants. | | | | | | | |  |
|  | | **Males (n=84)** | | **P value** | | **Females (n=162)** | | **P value** |  |
|  | | **Anxiety (n=42)** | **Healthy**  **(n=42)** |  | **Anxiety**  **(n=81)** | | **Healthy**  **(n=81)** |  |  |
|  | |  |  |  | |  |  |  |  |
| Age baseline (y) | | 15.7 | 15.7 | *p*= 1.000 | | 15.6 | 15.7 | *p*=.238 |  |
| **Education level** | |  |  |  | |  |  |  |  |
| Lower vocational & special education | | 5(12%) | 7(17%) | *p=.*564 | | 22(29%) | 12(16%) | *p=.*086 |  |
| Intermediate vocational | | 13(32%) | 9(22%) | *p=.*394 | | 15(20%) | 16(21%) | *p=.*858 |  |
| Higher vocational | | 9(22%) | 13(32%) | *p=.*394 | | 20(26%) | 17(23%) | *p=.*622 |  |
| Academic | | 14(34%) | 12(29%) | *p=.*695 | | 19(25%) | 31(40%) | *p=.*090 |  |
| **Anxiety severity** | |  |  |  | |  |  |  |  |
| YSR at baseline | | 0.3±0.29 | 0.1±0.21 | *p=*.019 | | 0.5±0.31 | 0.4±0.32 | *p*=.003 |  |
| ASR at follow-up | | 0.4±0.33 | 0.3±0.28 | *p*=.036 | | 0.6±0.39 | 0.4±0.33 | *p*<.001 |  |
|  | Legend. Reported values are means ± SD or n (%). N=sample size. y = years. YSR=Youth Self Report. ASR =Adult Self Report. | | | | | | | |  |

|  |  |  |  |  |  |
| --- | --- | --- | --- | --- | --- |
| ***Table S5.*** | | | | | |
| Variables predicting anxiety at follow-up in male and female adolescents, ranked by absolute value of standardized coefficients (highest to lowest). | | | | | |
| **Predictor Variables**  **Males** | | **Standardized Coefficient (β)** | **Unstandardized Coefficient (B)** | **Std. Error** | **p value** |
| Baseline Anxiety | | 0.399 | 0.510 | 0.072 | <0.001*** |
| Loudness Falling Slope (mean) | | -0.357 | -0.089 | 0.026 | <0.001*** |
| Loudness Peaks (per second) | | 0.340 | 0.158 | 0.039 | <0.001*** |
| Loudness Rising Slope (SD) | | 0.292 | 0.270 | 0.086 | 0.002** |
| F1 bandwidth (CV) | | -0.206 | -1.938 | 0.819 | 0.019* |
| F2 bandwidth (CV) | | 0.193 | 1.192 | 0.536 | 0.027* |
| logRel F0-H1-A3 (mean) | | -0.113 | -0.012 | 0.006 | 0.084 |
| SlopeV500-1500 (mean) | | 0.108 | 10.697 | 6.394 | 0.096 |
| F3 frequency (CV) | | 0.105 | 2.519 | 1.505 | 0.095 |
| **Predictor Variables**  **Females** | |  |  |  |  |
| LogRel F0-H1-A3 (mean) | | 0.613 | 0.077 | 0.017 | <0.001*** |
| Baseline Anxiety | | 0.448 | 0.501 | 0.057 | <0.001*** |
| Voiced Segments (per second) | | 0.442 | 0.256 | 0.057 | <0.001*** |
| LogRel F0-H1-A3 (CV) | | 0.378 | 0.362 | 0.110 | 0.001** |
| Jitter (mean) | | 0.318 | 0.392 | 0.168 | 0.021* |
| F0 (SD) | | -0.286 | -0.253 | 0.152 | 0.098 |
| F1 Bandwidth (CV) | | 0.244 | 3.110 | 1.102 | 0.005** |
| MFCC1V (mean) | | -0.232 | -0.037 | 0.015 | 0.012* |
| F3 Bandwidth (mean) | | 0.226 | 0.001 | 0.001 | 0.008** |
| F3 Amplitude (mean) | | -0.226 | -0.003 | 0.001 | 0.015* |
| MFCC1 (CV) | | -0.225 | -0.629 | 0.239 | 0.009** |
| F1 Bandwidth (mean) | | -0.197 | -0.002 | 0.001 | 0.098 |
| Loudness Rising Slope (SD) | | -0.192 | -0.164 | 0.074 | 0.028* |
| F2 Frequency (CV) | | -0.183 | -4.837 | 2.173 | 0.027* |
| logRel F0-H1-H2 (mean) | | -0.175 | -0.022 | 0.011 | 0.035* |
| MFCC4 (CV) | | -0.169 | -0.119 | 0.056 | 0.036* |
| Slope UV 0-500 (mean) | | -0.163 | -4.463 | 2.075 | 0.032* |
| F0 range (20^th^ to 80^th^) | | -0.155 | -0.076 | 0.053 | 0.154 |
| Loudness Falling Slope (SD) | | 0.151 | 0.158 | 0.092 | 0.088 |
| F1 Frequency (CV) | | 0.140 | 1.283 | 0.769 | 0.097 |
| Shimmer (mean) | | -0.126 | -0.659 | 0.457 | 0.150 |
| F0 Falling Slope (mean) | | -0.121 | -0.001 | 0.001 | 0.078 |
| F0 Rising Slope (mean) | | -0.088 | -0.000 | 0.000 | 0.171 |
| Legend. Std. Error = Standard error. ***Indicates significance at the level p<.001, ***p*<.01, *p<.05. | | | | | |

| ***Table S6.*** | | | | |
| --- | --- | --- | --- | --- |
| Acoustic-prosodic features selected for the longitudinal classification of Anxiety Disorder (AD) and Social Anxiety Disorder (SAD) in males and females. | | | | |
|  | **Males (AD)** | **Males**  **(SAD)** | **Females**  **(AD)** | **Females (SAD)** |
| F0 (mean) |  |  | 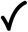 | 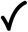 |
| F0 (SD) |  |  | 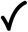 |  |
| F0 Falling Slope (mean) | 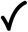 | 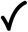 |  |  |
| F0 Falling Slope (SD) | 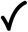 | 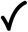 | 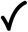 |  |
| F0 Rising Slope (mean) |  |  | 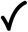 |  |
| F0 Rising Slope (SD) |  |  |  |  |
| F0 range (20^th^ to 80^th^) |  | 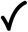 |  | 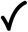 |
| F0 (20^th^ percentile) |  |  | 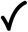 | 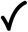 |
| F0 (50^th^ percentile) | 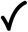 | 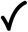 | 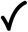 |  |
| Loudness (mean) |  | 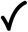 |  |  |
| Loudness (SD) |  |  |  | 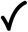 |
| Loudness Falling Slope (mean) | 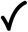 |  |  |  |
| Loudness Falling Slope (SD) |  |  |  |  |
| Loudness Rising Slope (mean) | 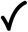 |  | 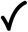 |  |
| Loudness Rising Slope (SD) |  |  |  | 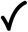 |
| Loudness range (20^th^ to 80^th^) |  |  |  |  |
| Loudness (20^th^ percentile) |  | 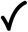 |  |  |
| Loudness (50^th^ percentile) |  | 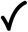 |  |  |
| Loudness (80^th^ percentile) |  | 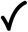 |  |  |
| Loudness peaks (per second) | 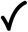 |  |  |  |
| F1 Frequency (mean) |  |  |  | 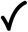 |
| F1 Frequency (SD) |  |  | 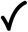 | 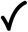 |
| F1 Amplitude (mean) | 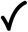 | 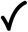 |  |  |
| F1 Amplitude (SD) |  | 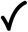 |  |  |
| F1 Bandwidth (mean) | 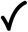 |  |  |  |
| F2 Frequency (mean) |  |  | 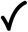 |  |
| F2 Frequency (SD) |  |  | 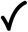 |  |
| F2 Amplitude (mean) | 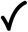 | 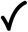 |  |  |
| F2 Amplitude (SD) |  | 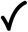 |  |  |
| F2 Bandwidth (mean) |  | 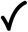 |  |  |
| F2 Bandwidth (SD) |  | 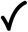 | 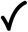 |  |
| F3 Frequency (mean) |  |  | 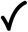 | 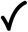 |
| F3 Amplitude (mean) |  | 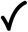 |  | 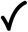 |
| F3 Amplitude (SD) | 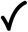 | 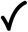 |  | 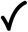 |
| HNRdBACF (mean) | 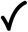 |  | 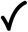 |  |
| Jitter (mean) |  |  | 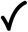 |  |
| Jitter (SD) |  |  | 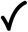 | 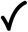 |
| Shimmer (SD) |  |  |  | 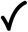 |
| Slope V0-500 (mean) | 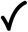 |  |  | 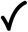 |
| Slope V 0-500 (SD) | 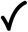 |  | 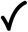 | 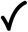 |
| Slope V 500-1500 (SD) | 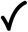 |  | 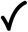 |  |
| Slope UV 0-500 (mean) | 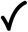 |  |  | 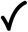 |
| Slope UV 500-1500 (mean) |  | 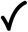 |  | 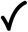 |
| Spectral Flux (mean) | 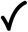 |  | 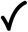 |  |
| Spectral Flux (SD) |  |  | 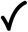 |  |
| Spectral Flux V (mean) |  |  | 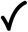 |  |
| Spectral Flux V (SD) |  |  | 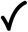 |  |
| Spectral Flux UV (mean) |  | 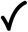 | 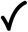 |  |
| Alpha Ratio V (mean) | 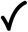 | 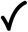 |  |  |
| Alpha Ratio V (SD) |  |  | 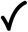 |  |
| Alpha Ratio UV (mean) |  |  |  |  |
| Hammarberg Index V (mean) |  | 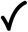 |  |  |
| Hammarberg Index V (SD) |  | 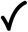 |  |  |
| Hammarberg Index UV (mean) |  |  |  | 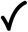 |
| logRelF0.H1.A3 (SD) |  |  | 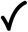 |  |
| MFCC1 (mean) |  | 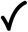 | 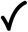 |  |
| MFCC1 (SD) |  |  |  | 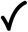 |
| MFCC1 V (mean) |  | 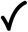 |  |  |
| MFCC1 V (SD) |  |  |  |  |
| MFCC 2 (mean) | 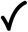 |  |  | 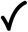 |
| MFCC 2 (SD) |  |  | 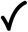 | 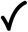 |
| MFCC 2 V (mean) | 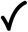 |  |  |  |
| MFCC 2 V (SD) | 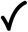 |  |  |  |
| MFCC 3 (mean) |  |  |  | 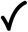 |
| MFCC 3 (SD) |  |  |  | 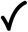 |
| MFCC 3 V (mean) |  |  |  |  |
| MFCC 4 (SD) | 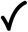 |  | 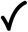 | 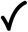 |
| MFCC 4 V (SD) | 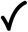 |  |  |  |
| Unvoiced Segment Length (mean) |  |  |  |  |
| Unvoiced Segment Length (SD) |  |  |  |  |
| Voice Segments per second |  |  |  |  |
| Voiced Segment Length (mean) |  |  |  |  |
